# First principles investigation of arsenic functionalized MgO nanoribbons

**DOI:** 10.1038/s41598-026-39119-w

**Published:** 2026-02-20

**Authors:** M. Sankush Krishna, Aruru Sai Kumar, Srinivas Kankanala, Anil Kumar Nayak

**Affiliations:** School of Electronics Engineering, VIT-AP University, Near AP Secretariat, Amaravathi, Andhra Pradesh 522241 India

**Keywords:** MgO nanoribbons, Density functional theory (DFT), Heavy metals, Non-equilibrium Green’s function (NEGF), Materials science, Nanoscience and technology, Physics

## Abstract

The variation in the properties of MgO Nanoribbons towards Arsenic (As) atoms is discussed in the current work. To evaluate the MgONRs behavior towards the As atoms, the first principles approach within the context of density functional theory is deployed to evaluate the electronic and transport characteristics of MgONRs. Results revealed that As-termination is found to improve the stability of the MgONRs compared to hydrogenated MgONRs (H–MgO–H). The electronic characteristics of MgONRs are significantly altered with As passivation. Further, the current–voltage (I–V) characteristics reveal a significantly enhanced current conductivity for the As-terminated MgONRs (As–MgO–As). This determines their transport characteristics are significantly enahnced with As termination. Further, the local device density of states showcase that the carrier transmission majorly occurs through the edges. From the acquired results, it can be concluded that MgONRs can be efficiently utilized as an effective material for the future nanoelectronic applications.

## Introduction

### 2D materials

The scaling of transistor technology has led to the advancement to the semiconductor industry. Recently, the semiconductor scaling has faced with the physical limitations owing to unwanted tunneling effects such as short channel effects, tunnelling leakage currents etc^1^. In view of this, the researchers are on the outlook for the potential alternatives that can continue the advancements of the semicodnctor technology^2^. Over the past few decades low-dimensional materials have been extensively investigated. In these categories of materials, confining the transport of electrons in certain directions has lead to dramatic changes in materials’ properties due to quantum confinement effects^3^. Two-dimensional materials confine physical phenomenon in a planar direction. A noticeable characteristics among the 2D materials is their size dependent properties^4^. Some semicondutor 2D materials exhibit a transitioning from indirect to direct bandgap as their thickness is reduced to monolayer size. This can enable enahnced performance in applications like photoluminescence^5^. In addition, at lower dimensions, the active surface sites and electronic conductivity can be significantly improved by reducing the vertical dimensions in 2D materials. This results in higher density of active sites improving the efficiency of energy conversion and storage like electrocatalysis and batteries^6,7^. The groundbreaking achievement in the domain of 2D materials has begun after the successful realisation of grahene from bulk graphite through mechanical exfoliation method. Graphene is a simple atomic thin sized planar sheet with hexagonally arranged carbon atoms^8^. Graphene exhibited some unique characteristics such as being the thinnest but mechanically the strongest material. In addition, a very high elecron mobility and thermal conductivity is noticed due to the linear crossing of conduction and valence band edges^9,10^.

### Nanoribbons

The as synthesized graphene is found to be a zero bandgap material. Consequently, a bandgap can be introduced in the graphene through methods like doping, straining and carving the nanoribbons out of graphene called graphene nanoribbons (GNRs)^11^. While 2D materials confine the physical effects in a plane, their quasi-1D allotropes such as nanoribbons futher confine to one direction. The charge carriers or excitons in the nanoribbons have one degree of freedom^12^. These materials are of atmost interest due to their exceptional properties such as higher electronic density of states, increased exciton binding energy, width dependent bandgaps, edge configuration dependent electronic properties, enahnced surface electronic or photon scattering^13^. Nanoribbon materials with thickness of single layer or few-atomic layers are quasi-1D allotropes of 2D materials and are potential candiates for investigation due to their size dependent fundamental properties^14^. After the synthesis of multiple 2D materials their nanoribbon allotropes attained prominence due to their requirement in nanoscale applications^15^. In this regard, various 2D materials beyond graphene such as mono-chalcogenides (SnSe, ZnSe, GaS, and GaSe), transition metal dichalcogenides (TiSe_2_, ReS_2_, WS_2_, MoS_2_), group IV (graphene, silicene), group III-V binary compounds, tri-chalcogenides (ZrS_3_, TiS_3_), black phosphorous and supercapacitors are investigated^16^.

### 2D MgO

Metal oxides like magnesium oxide, manganese oxide, ferrte oxide, zinc oxide are well recognized as an effective materials for sensors, insulators etc.^17–21^. In this context, magnesium oxide (MgO) is an alluring material owing to its fascinating opto-electronic and magnetic properties^22,23^. Both polarzied and non-polarized 2D MgO structures were synthesized by multiple researchers^24,25^. Non-layered 2D MgO nanosheets have been synthesized by Liu et al.^25^ using the two step process. In their experiment, the researchers have used Mg(OH)$_2$ as their precursor. Graphitic sheets of MgO with Mg and O atoms organized into a planar hexagon like pattern are successfully synthesized on gold and silver substrates^26,27^. Polar 2D MgO structures are highly intriguing because of their captivating magnetic and electronic characteristics^22,23,28,29^. 2D MgO nanoribbons (MgONRs) are theoretically examined in previous studies^30^. The flexibility of tailoring MgONRs’ characteristics with chemical modifications make them more interesting. MgONRs are investigated for their promising applications in nanoscale interconnects design with edge chemical modifications ^31,32.^ Recently, ZnO nanoribbons are used as heavy metal sensors^33^. However, the research efforts towards the MgONRs are still low.

The current manuscript presents the theoretical studies on As functionalized MgONRs. To conduct theoretical investigations on MgONRs, density functional theory (DFT) in conjunction with non-equilibrium Green’s function (NEGF) is employed. The edges of the MgONRs are terminated using As-atoms (As-MgO-As). To assess the influence of As functionalization, the study investigates the deviations in the electronic characteristics of the MgONRs. The bandstructures of the MgONRs are significantly altered after passivation with As, showing their sensitivity towards As-atoms. Additionally, the transport characteristics are assessed in order to quantify the MgONRs reaction to the As atoms. The conductivity of the MgONRs is significantly higher than that of the hydrogenated MgONRs (H-MgO-H). The results indicate that MgONRs have the potential as a suitable electrode material for designing nanosensors and future nanoelectronics applications.

The structure of this manuscript follows with “Computational method” describing the computational models deployed following the discussion on the obtained results in “Results and discussion”. Concluding remarks are provided in “Conclusion”.

**Fig. 1:**
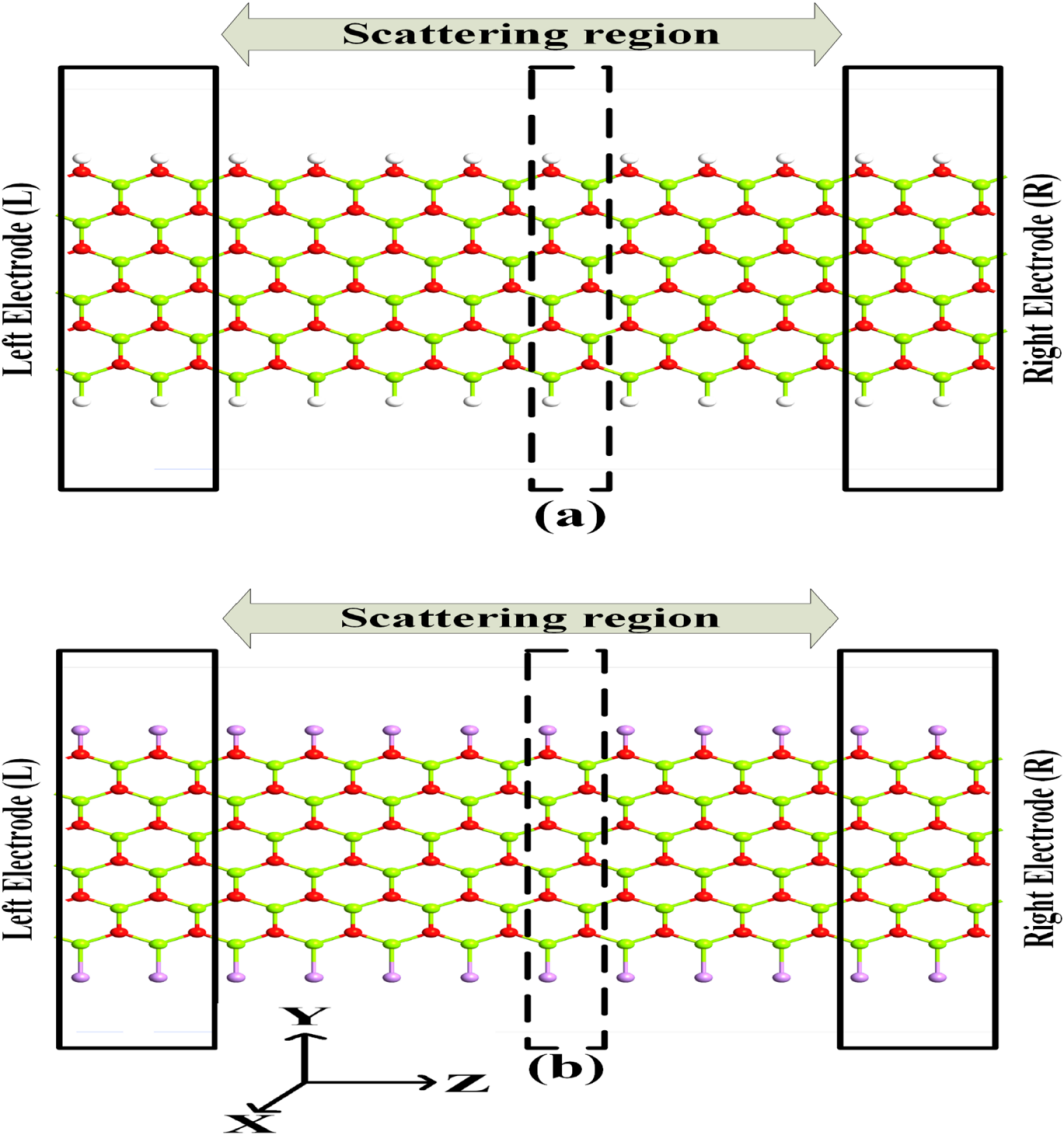
Two-probe structures of MgONRs (**a**) H–MgO–H, (**b**) As–MgO–As. Here, H, O, Mg and As atoms are represented by white, red, green, and purple balls, respectively. Unit cell is represented using the dashed line at the center.

## Computational method

The calculations in this work are all done in the QuantuWise ATK tool employing DFT and the NEGF method^34^. The supercell approach is used to optimize the considered MgONR structures. We repeat the nanoribbon unit cells in one direction (Z-axis) with periodic conditions, and confine them in the other two directions (X- and Y-axes). The structures are allowed to relax until the inter-atomic forces reach a value lower than 0.05 eV/Å. All MgONR configurations are optimized using a Monkhorst-pack k-point meshing of 1 $\times$ 1 $\times$ 101 in the X, Y, and Z directions, respectively. Optimized mesh cut-off energy about 150 Ry is used. Interferences from periodic images is suppressed by implementing a vacuum spacing of 10 Å. The exchange-correlation effects in all computations are modeled using the generalized gradient approximation (GGA) within the Perdew-Burke-Ernzerohf (PBE) functional. Double-zeta polarized basis sets have been used for the atoms. The energetic stability and the As-interaction stability has been determined by computing the binding energy ($E_b$) formation energy ($E_{form}$) and $E_{ads}$ using following relations^35–37^

1 $$\begin{aligned} E_{b}=\frac{1}{N_{T}}(E_T-pE_{Mg}-qE_O-rE_P) \end{aligned}$$

2 $$\begin{aligned} E_{form}=\frac{1}{N_T} (E_T-E_{bare}-n\mu _P) \end{aligned}$$

3 $$\begin{aligned} E_{ads}=\frac{1}{N_T} (E_T-E_{bare}-nE_{As}) \end{aligned}$$

The total energy of the MgONRs that are being studied is represented by the symbol $E_T$, total energy of the bare MgONR is represented by $E_{bare}$. Magnesium, oxygen, hydrogen, and arsenic atoms’ isolated energies are designated by the symbols $E_{Mg}$, $E_O$, $E_H$, and $E_{As}$, respectively. The $\mu _P$ represents the chemical potential of the passivated foreign atom. In order to compute the transport characteristics, the nanoribbons are modeled using the two-probe device configurations. The model consists of a central area or the channel area attached with electrodes on either side. In this method the current is computed using the Landauer-B$\ddot{\textrm{u}}$ttiker formula, which is shown below^38^,

4 $$\begin{aligned} I(V)=\frac{2e^2}{h}\int _{\mu _L}^{\mu _{R}}T(E,V)[F(E-\mu _L)-F(E-\mu _R)]dE \end{aligned}$$

The term *T*(*E*, *V*) denotes the coefficient of transmission, which is determined using the equation provided in Eqn. 5. The Fermi energy levels for the left and right electrodes are indicated by $\mu _{(L)}$ and $\mu _{(R)}$, respectively.

5 $$\begin{aligned} T(E,V)=T_r[\tau _R(E,V)G_C(E,V)\tau _L(E,V)G_C^+(E,V)] \end{aligned}$$

where, the retarded Green’s function is denoted by $G_C(E,V)$ and $G_C^+(E,V)$ denotes the advanced Green’s function. The left electrode coefficient of coupling is denoted by $\tau _L(E, V)$ and the right electrode coupling coefficient is designated by $\tau _R(E, V)$.

## Results and discussion

The subsequent part presents a detailed analysis of the MgONRs, based on the simulation results obtained. For better understanding, it is subdivided into structural, electronic, and transport properties.

### Structural properties

**Fig. 2:**
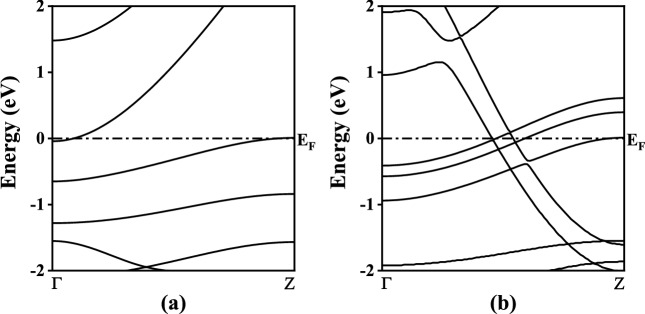
The computed MgONR bandstructures (**a**) H–MgO–H, (**b**) As–MgO–As. Dash dotted line at 0 eV represents Fermi energy level.

Firstly, to investigate the geometric properties of the nanoribbons, the nanoribbons are relaxed to attain the ground state. The relaxed atomic structures of the MgONRs are presented in Fig. 1 . In our calculation, Mg-O bond length is found to be 1.93 Å. This is approximately same as the values reported in earlier works^31^. The edge Mg-H and O-H bond lengths are 1.78 Å and 0.97 Å, respectively. However, upon As passivation, slight changes in these parameters can be noticed at the edges. The bond lengths at the edges are reduced for the As-MgO-As with measured Mg-O bond length at 1.92 Å at Mg-edge and 1.89 Å at O-rich edge. The As atoms bond with the edge Mg and O atoms with bond lengths of 2.63 Å and 1.90 Å, respectively. The higher bond lengths could be possibly due to the larger atomic size of arsenic atom.

In order to gain insigths on the stability of these nanoribbon structures, their binding energies are evaluated using Eq. (1). The obtained $E_b$ values for the pristine and As-terminated MgONRs are outlined in Table 1. Table 1 shows that the $E_b$ is negative for all the nanoribbons. The negative $E_b$ indicates the passivation is exothermic thereby stating that the structures are stable. The H–MgO–H structures have the $E_b$ value of -5.21 eV. In comparison, the As-MgO-As structures have relatively more negative $E_b$ of $-5.28$ eV. This points to the fact that the As-terminated strucures are more stable than the hydrognated structures. To further validate our findings we have evaluated the $E_{form}$ and $E_{ads}$ of the MgONR structures using the expression in Eqs. (2) and (3), respectively. The evaluated $E_{form}$ and $E_{ads}$ values are tabulated in Table 1. The $E_{ads}$ for the H–MgO–H is computed to be around $-0.62$ eV. It is improved to $-0.69$ eV for the As-MgO-As structures. The more negative $E_{ads}$ refers to the stronger binding of As atoms with the MgONRs. The $E_{form}$ of the H–MgO–H is computed to be at $-0.135$ eV. The negative $E_{form}$ indicates the practical feasibility of the structures. The $E_{form}$ of the As–MgO–As is found to be $-0.14$ eV. The more negative $E_{form}$ indicates the As–MgO–As structure as the more stable structure.

**Table 1:** The computed edge passivation bond lengths, binding energy ($E_b$), and energy band gap ($E_g$), adsorption energy ($E_{ads}$) and formation energy ($E_{form}$) of the M*g*ONRs.

Structure	Mg-rich edge (Å)	O-rich edge (Å)	$E_b$ (eV)	$E_g$ (eV)	$E_{ads}$ (eV)	$E_{form}$ (eV)
H–MgO–H	1.78	0.97	$-5.21$	Metal	$-0.62$	$-0.13$
As–MgO–As	2.63	1.90	$-5.28$	Metal	$-0.69$	$-0.14$

**Fig. 3:**
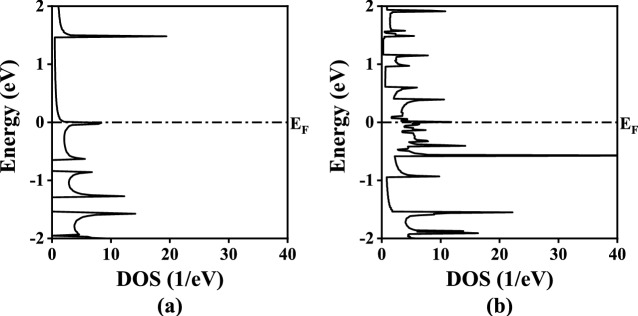
The computed MgONR DOS plots (**a**) H–MgO–H, (**b**) As–MgO–As. Dash dotted line at 0 eV represents Fermi energy level.

**Fig. 4:**
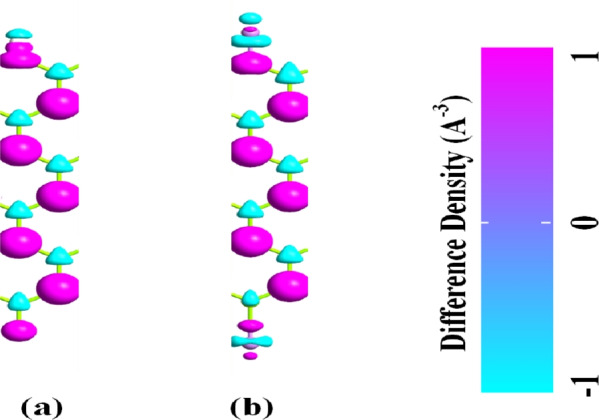
Charge difference density plots of (**a**) H–MgO–H and (**b**) As–MgO–As.

### Electronic properties

From the $E_b$ calculations it is evident that the structures are stable implying their practical feasibility. Now to check the behavior of MgONRs towards the As atoms, the electronic characteristics are investigated using the bandstructure and density of states (DOS) calculations. The results for the bandstructure and DOS analyses are presented in Figs. 2 and 3, respectively. The MgONRs have energy states cutting through Fermi energy level ($E_F$). This is an indication of the metallic behavior in the structures. One can see that the energy states in the bandstructure are altered due to the As functionalization. Upon As passivation, the bandstructure is significantly changed indicating its sensitivity towards As atoms. Interestingly, the metallic nature is still retained. The significant changes to the bandstructure occur due to mutual interactions of edge MgO atoms with foreign As atoms.

**Fig. 5:**
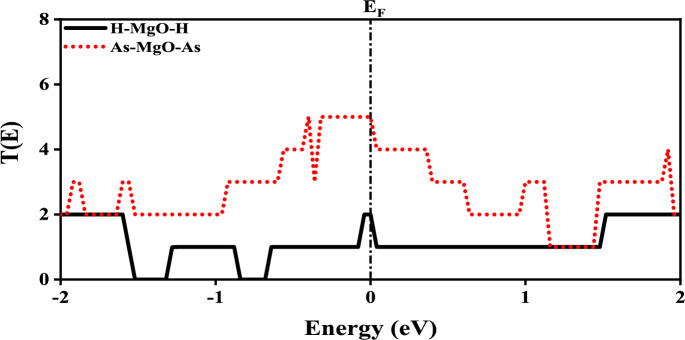
Zero bias transmission spectrum plots of MgONRs.

**Fig. 6:**
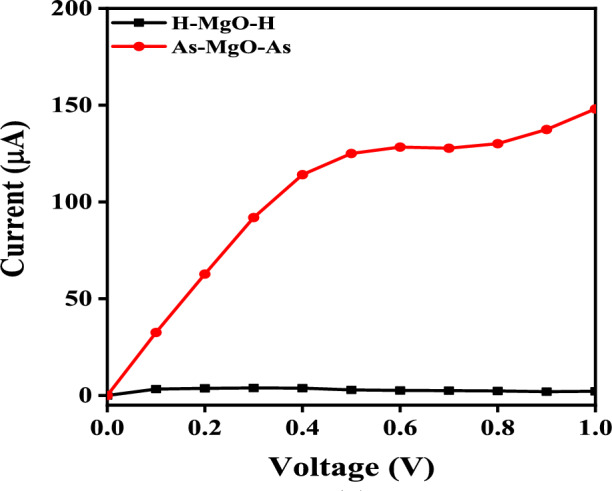
I-V characteristics of considered MgONR devices.

Further, the DOS profiles are shown in Fig. 3. Similar to bandstructure results, non-zero DOS at the $E_F$, indicates a metallic behavior of the MgONR structures. In the conduction band, the energy states arise due to the interactions of oxygen and hydrogen atoms. Below the $E_F$ energy level in the valence band, the energy states are a result of Mg-H interactions^31^. For the As–MgO–As nanoribbons, the major changes in the bandstructure at the $E_F$ is due to the As passivation. The DOS states are higher in valence band which is in accordance with the bandstructure plots in Fig. 2b.

**Fig. 7:**
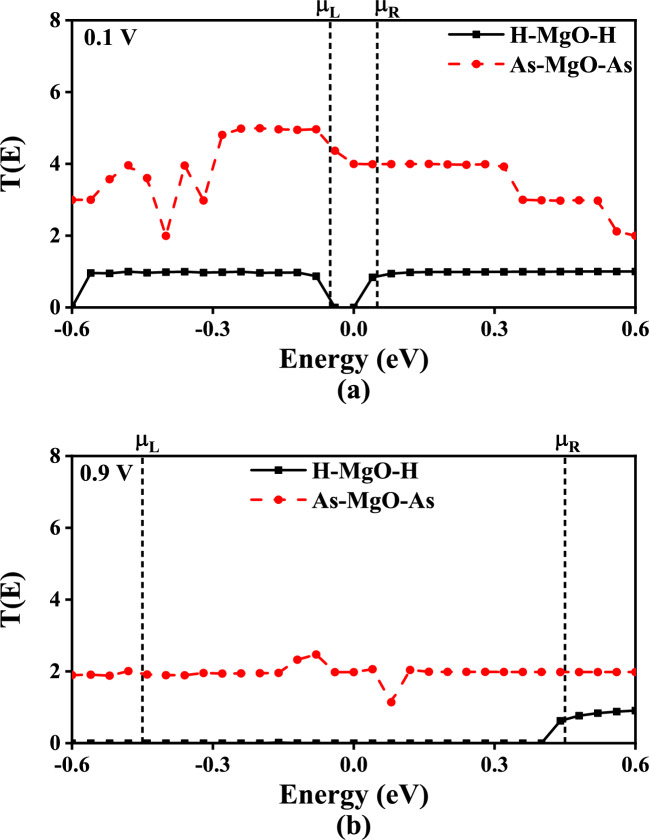
Bias dependent transmission spectrum plots of the As-passivated MgONRs at (**a**) 0.1 V and (**b**) 0.9 V.

To further comprehend the interaction of atoms in the MgONRs, we have evaluated the charge difference density (CDD) characteristics. The CDD plots of the considered MgONR structures presented in Fig. 4. From the figure, two types of charge distribution can be observed. The positive color distribution is observed across the oxygen atoms whereas the negative charge color distribution is observed across the Mg atoms. The positive value indicates the charge accumulation. This implies that the charge is acquired by the oxygen atoms. For the regions with negative charge density, they define the charge depletion regions^39^. Since oxygen atoms being more electronegative they act as charge accumulators whereas the Mg atoms being electropositive atoms act as charge depletion regions. The charge transfers from the Mg atoms to the oxygen atoms. For the H-MgO-H at the O-rich edge, it can be noticed that hydrogen atoms act as charge depletion regions. However, at the Mg-rich edge the H atoms act as charge accumulators. This is due to H atoms being more electropositive than the Mg atoms. For the As-MgO-As nanoribbon at the O-edge, the Arsenic atoms act as the charge depletion regions whereas at the Mg-rich edge they act as the charge accumulators. Moreover, the Mulliken population analysis is performed to evaluate the charge transfer at the edges. For the As-MgO-As nanoribbon, a charge transfer of 0.155*e* is noticed from As atoms to the oxygen atoms. At the Mg-edge, a charge transfer of 0.91*e* is noticed from the Mg atom to the As atom.

**Fig. 8:**
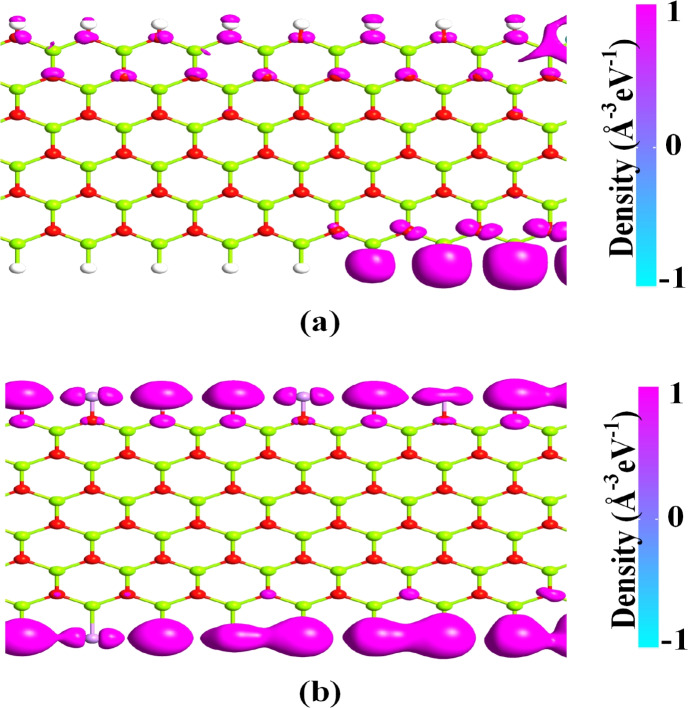
LDOS plots of (**a**) H–MgO–H and (**b**) As–MgO–As at 0.9 V.

### Transport properties

From the electronic properties investigation, the As-terminated MgONRs are found to be metallic with significant variations in bandstructures. To further evaluate the impact of As-termination at the edges of MgONRs, the transport characteristics are evaluated. To examine the transport characteristics of the considered MgONRs two-probe model is deployed. The approach involves interfacing periodic electrodes on either sides. For the considered MgONR devices, the transmission spectrums are evaluated at an applied potential of 0 V and presented in Fig. 5. The transmission spectrum peak for As–MgO–As is more than twice the transmission peak of H–MgO–H. The increase in transmission peak is in accordance with the increased DOS peak for As–MgO–As (Fig. 3a). This determines that the As-passivation has improved the carrier transport probability.

Further, the evaluated MgONR devices’ I–V results are presented in Fig. 6. It can be observed that the current magnitude of H-MgO-H is significantly low when compared to As–MgO–As. The larger current magnitude in As-MgO-As could be due to the edge MgO atoms interactions with the As atoms. The current rises in a linear fashion until it reaches 0.4 volts, but begins to deviate from this linear trend after 0.5 volts. This drastic variation in the current magnitude of As–MgO–As shows that As termination at the edges significantly enhances its transport behavior.

To understand the MgONRs behavior towards the As atoms, the transmission spectrum with respect to the bias voltage are evaluated and depicted in Fig. 7. As can be observed from the Fig. 7a at 0.1 V, the integral area of the transmission curve within the electrode electrochemical potentials ($\mu _{L/R}$) is higher for the As–MgO–As. This further explains the increased current magnitude in As–MgO–As. This indicates the MgONRs are highly sensitive towards the As atoms. However, as the bias voltage is increased to 0.9 V, the integral area is significantly increased for the As–MgO–As. For H-MgO-H a slight reduction is noticed. Due to increased transmission area at higher bias voltage, the current magnitude increases for As–MgO–As whereas for the H–MgO–H it is reduced. Consequently the transport characteristics of the nanoribbons are enhanced for As–MgO–As, which can be observed from Fig. 6.

To further validate our results the local device DOS (LDOS) is evaluated at 0.9 V and presented in Fig. 8. Figure 8a represents the LDOS of pristine MgONR device whereas for the As–MgO–As device it is depicted in Fig. 8b. For the As–MgO–As the energy states are densely located at the edges across the central region when compared to H–MgO–H device. The higher density of states across the edges increases the probability of carrier transport across the edges which further validates the higher magnitude of current in As–MgO–As compared to H–MgO–H device. For As–MgO–As the density is higher at the Mg-edge which signifies higher transmission of charge carriers across the Mg-edge.

### Limitations

Although our results depict a computational analysis of MgONR behavior towards As atoms, our work still has few limitations. The theoretical investigations still require experimental validation. In our work we have considered a defect free MgONR. The practical synthesis of material can cause many changes in the material electronic characteristics due to defects^40^. Also, the ambient factors like humidity, pH etc., also effect the transport characteristics of the As terminated MgONRs. However, we hope our theoretical investigation could motivate the future experimentalists to perform practical analysis on MgONRs with As functionalization.

## Conclusion

In this work, the theoretical investigations of MgONRs are done using the DFT to explore electronic transport behavior towards the As atom functionalization. The MgONR has been functionalized with As at the edges. The nanoribbons’ stability has been analyzed through the $E_b$ computations. Results infer that the Arsenic has relatively improved the stability compared to hydrogenated nanoribbons. The electronic properties confirms the As-termination resulted in significant variation of its bandstructure. In addition, to confirm and quantify the detection ability of the MgONRs, the quantum transport in terms of I-V characteristics are analyzed. Transport characteristics reveal a significantly enhanced current magnitude for As-MgO-As devices.Based on the results, it can be concluded that MgONRs have the potential to be used for nanoelectronic applications. The edge chemistry dependent transport behavior shows their significant potential as the nano electrode or contact material in nanoscale sensing devices.

## Data Availability

The datasets used and/or analysed during the current study available from the corresponding author on reasonable request.
